# Asymmetrical chromosomal utilization during zygotic genome activation

**DOI:** 10.3389/fbinf.2026.1851810

**Published:** 2026-07-01

**Authors:** Lily Acker, Chuan Qin, Hui Chen

**Affiliations:** Department of Biological Sciences, University of South Carolina, Columbia, SC, United States

**Keywords:** chromosome activity, early embryogenesis, genome evolution, maternal-to-zygotic transition, nascent transcription, subgenome, *Xenopus*, zygotic genome activation

## Abstract

Following fertilization, the early embryonic development requires zygotic genome activation (ZGA), during which hundreds to thousands of genes are transcribed from an initially dormant genome. However, how individual chromosomes are differentially utilized during this critical process remains largely unknown. Here, we investigate chromosome-specific transcriptional activity during ZGA in *Xenopus laevis*, an allotetraploid organism containing long (L) and short (S) subgenomes. Using the temporal nascent transcriptome data generated by 5-ethynyl uridine (5-EU) metabolic labeling and sequencing, we calculated the activation index (AI) for each chromosome and uncovered striking differences in transcriptional activity and relative contributions to ZGA. We discovered differential ZGA activities both at the chromosomal level and at the subgenome level, with a general higher activity in the L subgenome than the S subgenome. Moreover, while both L and S subgenomes show spatially heterogeneous ZGA activity within territories of chromosomes, the L subgenome territories show higher ZGA activity than the S subgenome, correlated with the regional gene density. Importantly, inhibition of protein translation at early developmental stages selectively abolished transcription from the most active, primarily L chromosomes without equivalently affecting their S counterparts or other chromosomes, suggesting that the maternal translational products amplify the ZGA output of specific chromosomal territories as units, not merely the expression of individual ZGA genes on those chromosomes. Together, our findings reveal an asymmetrical pattern of chromosomal utilization that shapes the landscape of ZGA during early embryogenesis.

## 1 Introduction

In the earliest stages of development, a zygote relies on maternal products to direct cellular function, as the genome is not yet active. However, once the zygote exits these preliminary stages, it undergoes a process known as the maternal-to-zygotic transition (MZT), which hands off developmental control from mother to zygote (Tadros and Lipshitz, 2009; Vastenhouw et al., 2019). This MZT involves the coordination of maternal clearance, or degradation of maternal transcripts, and zygotic genome activation (ZGA), when the embryo’s genome first produces nascent transcripts essential to subsequent development (Schier, 2007; Tadros and Lipshitz, 2009; Lee et al., 2014; Jukam et al., 2017; Vastenhouw et al., 2019). Despite some variations in timing or identity of genes expressed, ZGA remains highly conserved across species, which highlights how crucial this process is to early development (Li et al., 2013; Jukam et al., 2017). Thus, understanding how ZGA is regulated is fundamentally important for revealing principles of early embryonic development, and may inform novel strategies for preserving species or improving reproductive health such as preventing infertility and treating congenital disorders.

Previous work on various model embryonic systems has uncovered several mechanisms regulating ZGA, as has been extensively reviewed (Schulz and Harrison, 2019; Wu and Vastenhouw, 2020; Zhou and Heald, 2023; Kojima et al., 2025). These mechanisms converge on the embryo’s reprogramming its initially dormant genome into a state that is transcriptionally competent, primarily through titration of transcriptional repressors, translation of transcriptional activators, and remodeling of chromatins (Schulz and Harrison, 2019; Kojima et al., 2025). However, the majority of these studies focus on the ZGA regulation at the gene or whole embryo level; how individual chromosomal activity contributes to ZGA is not well understood. Addressing this gap may provide new mechanistic insights into genome regulation at the chromosomal level in early embryonic development.

One major challenge in determining individual chromosomal activity during ZGA is measuring authentic level of transcriptions from each chromosome, as maternal transcripts can mask the detection of newly produced transcripts, particularly those transcribed from lowly activated genes (Schier, 2007). Several methodologies have recently been developed to biochemically purify and quantify the nascent transcriptome from early embryos, including using 5-ethynyl uridine (5-EU) (Chan et al., 2019; Chen et al., 2019; Kwasnieski et al., 2019; Chen and Good, 2022) or 4-thio-uridine (4-sU) (Heyn et al., 2014; Bhat et al., 2023) to metabolically label and profile new transcripts. These approaches enable sensitive detection of zygotic transcription, including within individual chromosomes, thus providing further insight into the mechanisms of ZGA.

The model organism *Xenopus laevis* is uniquely suited to address the chromosomal contribution to ZGA, particularly from an evolutionary perspective, as they are allotetraploids with two homoeologous subgenomes acquired from ancestral diploid hybridization. Although they share over half of their genes, these genomes have evolved asymmetrically; the Long (L) genome has preserved more of the ancestral genomic content, while the short (S) genome has undergone higher levels of gene rearrangement or loss (Session et al., 2016). Despite this, comparisons with diploids *Xenopus tropicalis* or zebrafish reveal overall conservation of gene expression when homeolog contributions are considered together (Session et al., 2016; Phelps et al., 2023). This suggests a strong selective pressure to maintain a ‘core’ transcriptional program and supports *Xenopus laevis* as an appropriate model for investigating ZGA on a chromosomal level.

In this study, we quantitatively assessed the activities of individual chromosomes and chromosomal regions during ZGA by analyzing the temporal nascent transcriptome using EU-RNA-seq in early *Xenopus laevis* embryos (Chen and Good, 2022). By calculating the activation index (AI) for each chromosome, we were able to directly compare the chromosomal activity between the subgenomes and discover an asymmetrical utilization of chromosomes during ZGA. Further, we found that protein translation could be critical for regulating chromosome-specific utilization in ZGA. Our findings reveal a previously unknown mechanism of ZGA at the chromosomal level, reflecting higher-order genome regulation in early embryogenesis.

## 2 Methods

### 2.1 Data preparation

The collection of the *Xenopus laevis* nascent transcriptome data used in this study was described previously (Chen and Good, 2022), and the data were deposited in the Gene Expression Omnibus (GEO) https://www.ncbi.nlm.nih.gov/geo/. The datasets from the EU-RNA-seq for embryos (two biological replicates) at 5, 6, 7, 8, and 9 h post-fertilization (hpf) (GSE201835), which covers stages from pre-ZGA to post-ZGA, were used for analyzing chromosomal activity during ZGA. The sequencing data in fastq files were aligned to *Xenopus laevis* genome build 9.2 (Xenbase) and the transcript reads were quantified using salmon v0.12 (Patro et al., 2017). Briefly, the reference XENLA9.2 transcriptome fasta file was downloaded from Xenbase and used for index by using “salmon index -p 6 -t [XENLA9.2 transcriptome fasta file] -i [Salmon index file] -k 31”. Transcript reads from the sequencing result fastq files were aligned and counted by using “salmon quant -p 8 -i [Salmon index file] -l A [fastq files] --validateMappings -o [output reads file]”. The reads were further normalized by the sequencing depth using DESeq2 (Love et al., 2014).

### 2.2 Quantification of authentic nascent transcripts

To eliminate the potential effect of nonspecific binding of transcripts to beads during EU-RNA purification (Chen and Good, 2022; Chen, 2025), for each gene, the mean reads of the replicates were used for analysis and the net increase in reads (i.e., delta reads) at 6-9 hpf were calculated by Equation 1 using the reads at 5 hpf (pre-ZGA) as a baseline.

$${\Delta Reads}_{t}={Reads}_{t}-{Reads}_{5\;hpf},t\in \left\{6,7,8,9\right\}$$ (1)

where ${Reads}_{t}$ is the number of reads at developmental time t (6, 7, 8, and 9 hpf) and ${Reads}_{5\;hpf}$ is number of reads at 5 hpf (baseline).

To visualize the new transcription on each chromosome, the delta reads for all genes were mapped into individual chromosomes and represented as spikes. The relative contribution of each chromosome to the transcriptional output at each stage was mapped in a chord diagram for visualization.

### 2.3 Comparing chromosomal activity

The activation index (AI) for each chromosome at each stage was represented as the total delta reads per kilobase pair (kb), as defined by Equation 2.

$${AI}_{c,t}=\frac{\sum {\Delta \text{Reads}}_{c,t}}{{\text{Length}}_{c}\;\left(\text{kb}\right)}$$ (2)

where ${AI}_{c,t}$ denotes the activation index for chromosome c at stage t , $\sum {\Delta \text{Reads}}_{c,t}$ represents total delta reads mapped to chromosome c at stage t , and ${\text{Length}}_{c}\;\left(\text{kb}\right)$ is chromosome length in kilobase pairs.

To compare the chromosomal activities during ZGA, the AI was used for hierarchical clustering using the complete linkage method and the clusters were visualized using pheatmap in R. The clustering was only performed for the rows (i.e., chromosomes), but not the columns (i.e., developmental stages). The AI L/S ratio for chromosome pairs was calculated by Equation 3 to compare the biased chromosome utilization, and the genetic L/S ratio was calculated by Equation 4 to examine the genetic effect on the AI.

$${AI}_{L/S}\left(t,c\right)=\frac{{AI}_{L}\left(t,c\right)}{{AI}_{S}\left(t,c\right)}$$ (3)

where ${AI}_{L/S}\left(t,c\right)$ indicates the AI ratio for the chromosome pair c at stage t , ${AI}_{L}\left(t,c\right)$ and ${AI}_{S}\left(t,c\right)$ represent the AI for the L and S subgenomes of the chromosome pair c at stage t , respectively.

$${GR}_{\frac{L}{S}}\left(c\right)=\frac{G_{L}\left(c\right)/{Length}_{L}\left(c\right)}{G_{S}\left(c\right)/{Length}_{S}\left(c\right)}$$ (4)

where ${GR}_{L/S}$ indicates the genetic L/S ratio for chromosome pair c at stage t , $G_{L}\left(c\right)/{Length}_{L}\left(c\right)$ and $G_{S}\left(c\right)/{Length}_{S}\left(c\right)$ represent the gene density per kilobase for e the L and S subgenomes of the chromosome pair c , respectively.

To characterize the intra-chromosomal heterogeneity in ZGA, first the transcriptional activity of each gene (i.e., gene slope) was calculated by fitting a linear model to the delta reads from 5 to 9 hpf (Equation 5), and then the number of actively transcribing genes within a range of 1 megabase pair (1 Mbp) from a specific gene was counted using a criteria of gene slope >1 (Equation 6).

$${\Delta Reads}_{g}\left(t\right)=\beta_{0,g}+\beta_{1,g}t$$ (5)

where: $\beta_{1,g}$ is gene slope (transcriptional activity) for gene g , t is developmental time from 5–9 hpf, and ${\Delta Reads}_{g}\left(t\right)$ is delta reads for gene g at time t . The gene is considered actively transcribing if $\beta_{1,g}$ > 1. The number of actively transcribing neighboring genes within 1 Mbp of a gene were calculated by:

$$A_{i}=\sum_{j\in N_{i}}\left(\beta_{1,j}\;>\;1\right)$$ (6)

where: $A_{i}$ is the number of active neighboring genes around gene i, $\beta_{1,j}$ is gene slope of each neighboring gene j located within the defined genomic window (e.g., ±1 Mbp from gene i ), and $N_{i}$ is the set of genes within ±1 Mbp of gene i .

The density of activated genes in individual chromosomes and the distribution of activated genes within 1 Mbp from a specific gene were visualized in heat maps (Figures 2A,B) and histograms by geom_histogram in ggplot2 with bin sizes of 30 (Supplementary Figure S2). The densities of activated genes for individual chromosomes (red lines in Supplementary Figure S2) and all chromosomes (dashed black lines in Supplementary Figure S2) were determined by geom_density function that computes and draws the kernel density estimate, a smoothed histogram. To characterize the landscape of the nascent transcription across all chromosomes, the delta reads from 5 to 9 hpf were binned at an interval of 1 Mbp for each chromosome (Equation 7), and the log_2_ slope for each bin was calculated as the rate of transcription in a specific binned region (Equation 8) (Figure 3).

$$B_{c,k}\left(t\right)=\sum_{g\in B_{c,k}}\left({\text{Reads}}_{g,t}-{\text{Reads}}_{g,5hpf}\right)$$ (7)

$$\log_{2}B_{c,k}\left(t\right)=\alpha_{0,c,k}+\alpha_{1,c,k}t$$ (8)

where $B_{c,k}\left(t\right)$ is the total delta reads in bin k of chromosome c at time t , $B_{c,k}$ is the set of genes located within the 1 Mbp bin, ${\text{Reads}}_{g,t}$ is the reads for gene g at time t , ${\text{Reads}}_{g,5hpf}$ is the reads for gene g at 5 hpf baseline, $\alpha_{1,c,k}$ is the log_2_ slope representing the transcription rate in that genomic region, and t is the developmental time from 5–9 hpf.

The correlation between gene density (represented by the numbers of genes in each bin of individual chromosomes) and regional ZGA activity (represented by log_2_ bin slope for each 1 Mbp bins) was determined by the cor. test function in R, and the Pearson’s correlation coefficient (r) and p value (p) were reported and visualized in the scatter plots. The kernel density of the distribution was determined by geom_density_2 d and shown as contour lines in the scatter plots.

### 2.4 Effect of cycloheximide (CHX) on chromosomal activity

To identify the effect of protein translation in chromosomal activity during ZGA, the datasets from the EU-RNA-seq for control- and cycloheximide (CHX)-treated embryos at 5 and 7.5 hpf (GSE201834) (Chen and Good, 2022) were used for analysis. The AI for each chromosome was calculated as described above.

### 2.5 Statistical analysis

The statistical significance between groups was determined by One-Way Analysis of Variance (ANOVA) using the aov function in R, followed by Tukey’s Honestly Significant Difference (HSD) using the TukeyHSD function in R. The adjusted p values were used to indicate the statistical difference. The correlation analysis was performed by using the Pearson method in the cor. test in R, and the Pearson’s correlation coefficient and p value were used to compare the statistical significance. Symbols used for the statistical significance: ****, p < 0.0001; ***, p < 0.001; **, p < 0.01; *, p < 0.05; ns, not significant.

## 3 Results

### 3.1 Differential subgenome activation during ZGA

**FIGURE 1:**
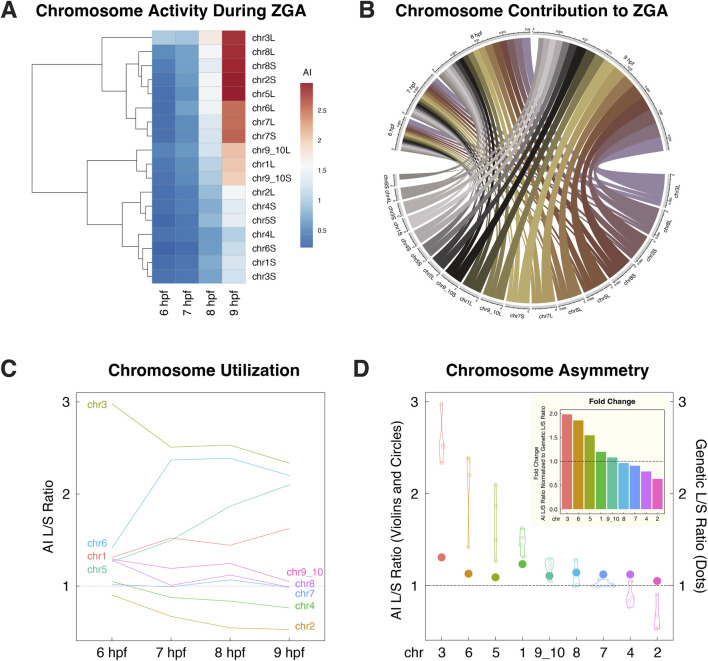
Asymmetrical Chromosome Utilization during Zygotic Genome Activation (ZGA). **(A)** Heatmap for the activation index (AI), an indicator of chromosomal activity during ZGA, for each chromosome in *Xenopus laevis* early embryos. Data shown are clustered using hierarchical clustering. The timepoints shown are embryonic stages from pre to post ZGA. **(B)** Chord diagram showing transcriptional contribution from each chromosome to each stage during ZGA. The numbers around the circles indicate the nascent transcripts per base pair from each chromosome, a proxy of the capacity of new transcriptional output from each chromosome during ZGA. **(C)** Line plot showing the ratio of AI for each L/S chromosome pairs at indicated developmental stages. Chromosomes names are labeled. Hpf, hours post-fertilization. **(D)** Violin plot showing the AI L/S ratio for specific pairs of chromosomes at 6-9 hpf. Each circle represents each time point. The solid dots indicate the genetic L/S ratio. The inset bar graph shows the fold change of the AI L/S ratio normalized to the genetic L/S ratio. The shown statistical symbols are based on comparison of the AI L/S ratio for each group with Chr 7 (AI L/S ratio ≈1). ****, p < 0.0001; *, p < 0.05. The statistical significance was determined by one-way ANOVA and Turkey’s HSD.

Previously, we metabolically labeled nascent transcripts with 5-EU in *Xenopus laevis* embryos from 5 (pre-ZGA) to 9 (post-ZGA) hours post-fertilization (hpf) cultured at room temperature and purified the nascent EU-RNA after biotinylating via click chemistry. In profiling the temporal dynamics of the nascent transcriptome during ZGA, we demonstrated a much higher sensitivity of the nascent EU-RNA-seq in measuring zygotic transcripts than conventional RNA-seq, enabling more accurate determination of ZGA onset (Chen and Good, 2022). To identify whether chromosome-specific transcriptional activity differs during ZGA, we quantified the net increase in nascent transcripts for each gene between 6-9 hpf relative to their pre-ZGA 5 hpf levels (i.e., delta reads), mapping these changes to each chromosome (Supplementary Figure S1). This allowed us to directly visualize the dynamics of nascent transcription across chromosomes and compare transcriptional activity across developmental stages and between chromosomes. As expected, we observed a continuous increase in transcription from 6 to 9 hpf for all chromosomes, suggesting that all chromosomes gradually become active as ZGA progresses (Supplementary Figure S1). To compare the transcriptional activity of chromosomes, we used the delta reads per kilobase pair (kb) as the activation index (AI) for each chromosome at specific stages (Supplementary Figure S1). This index reflects the capacity of transcriptional output from individual chromosomes and can be used as an unbiased proxy of chromosomal activity, regardless of chromosome sizes or gene positions. By comparing AI across chromosomes, we found that while AI increases from 6 to 9 hpf on each chromosome, differences in AI between chromosomes becomes more pronounced as ZGA progresses (Figure 1A; Supplementary Figure S1). This suggests differential transcriptional activities and dynamic regulation of chromosomes during ZGA.

To identify whether the differential chromosomal activity determines a chromosome’s contribution to ZGA, we calculated nascent transcripts per base pair from each chromosome and compared the relative transcription from each chromosome at each stage. We found that the chromosomal activity is largely proportional to the relative contribution to ZGA (Figure 1B). Notably, chromosome 3 L (chr3L) contributed most significantly to ZGA (Figure 1B) and is consistently more active than other chromosomes, starting from early stages (Supplementary Figure S1). This is potentially due to the early activation of key developmental genes on this chromosome, including nodal5 (xnr5) and nodal6 (xnr6), which have been shown to be expressed in embryos at the pre-midblastula transition (MBT) stages (Skirkanich et al., 2011). Thus, our AI analysis captures heterogeneous chromosomal activities and reveals their contribution to ZGA.

Next, we wondered whether the chromosomal activity differs in each subgenome pair (L vs. S), since they share conserved genes, albeit with altered organization and chromosomal size (Session et al., 2016). To test this, we compared the ratio of AI for each pair of L/S chromosomes. Strikingly, the majority of L chromosomes, particularly those of chr3 and chr6, show much higher activities than their S counterparts across the entire ZGA process, suggesting a biased utilization of the L subgenome in genome activation (Figures 1C,D). Moreover, we performed hierarchical clustering on the AI of all chromosomes across ZGA. Interestingly, we found that while some L/S pairs show the same (such as chr7L/7 S and chr8L/S) or similar activities (such as chr4L/4 S and chr9_10L/9_10 S) during ZGA, 2 L/S pairs (i.e., chr3L/3 S and chr6L/6 S) exhibit strikingly different activities: chr3L and chr6L are among the most active chromosomes, and chr3S and chr6S are among the least active chromosomes (Figure 1A; Supplementary Figure S1). To understand whether the L-biased utilization is influenced by the genetic bias, we calculated the genetic L/S ratio based on the gene numbers and chromosome sizes. We found that the genetic L/S ratio showed a minor variation between chromosomes (solid dots in Figure 1D). Further normalization of the AI L/S ratio to the genetic L/S ratio revealed similar trend of L-biased utilization to that of the AI L/S ratio alone (inset in Figure 1D). These data suggest that while the chromosomal gene content contribute to the baseline AI asymmetry, an additional layer of chromosome-scale regulatory or epigenetic control exists for the differential utilization of the suggenomes.

### 3.2 Intrachromosomal heterogeneity during ZGA

**FIGURE 2:**
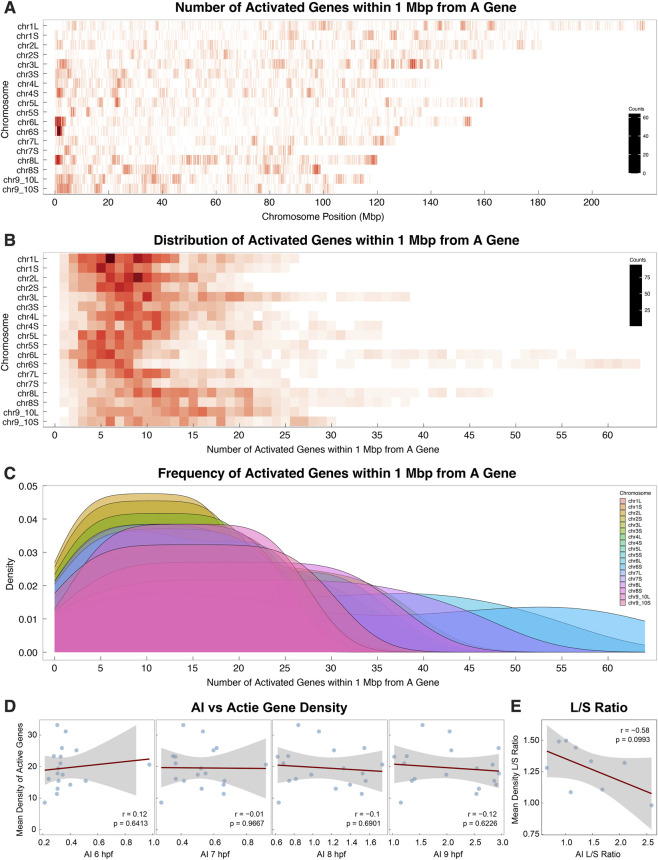
Intrachromosomal heterogeneity during ZGA. **(A)** Heatmap for the number of activated genes within a range of 1 million base pairs (Mbp) from a gene on each chromosome, from 5-9 hpf. The gene activity was determined by fitting a linear model and the genes with a slope >1 are included. **(B,C)** Distribution **(B)** and frequency **(C)** of activated genes within 1 Mbp from a gene on each chromosome. Data measured from **(A)**. **(D)** Scatter plots showing the correlations between chromosomal AI at indicated developmental stages and the mean density of active genes. **(E)** Scatter plot showing the correlation between the AI L/S ratio and the mean density L/S ratio between chromosomes. In both D and E, each dot represents each chromosome. The Pearson’s correlation coefficients (r) and p values are shown in individual plots. The redlines indicate the regression trend from a linear fitting, and the grey ribbons represent the confidence interval of the fitted trend.

Mapping nascent transcription on chromosomes revealed regions with both high and low active gene density (Supplementary Figure S1; Supplementary Figure S2A), suggesting nonuniform activation of individual chromosomes. We asked whether intrachromosomal heterogeneity accounts for the differential and asymmetrical subgenome utilization during ZGA. To address this, we calculated the slope of each gene activation by fitting a linear model to the temporal nascent transcriptome data and quantified the number of active genes (slope >1) within 1 Mbp regions across each chromosome (Figure 2A). This allowed us to measure the density of active subregions in chromosomes (Figures 2B,C; Supplementary Figure S2B). Interestingly, while each chromosome shows highly transcriptionally active regions (Figure 2A), several chromosomes, independent of size or position, contain a higher density of active genes, including chr6L, chr6S, chr8L, chr8S, and chr3L (Figures 2B,C). However, no correlation was found between the chromosomal AI and the density of active genes (Figure 2D). Further, a weak, but not significant, correlation was found between the AL L/S ratio and the mean density L/S ratio (r = −0.58, p = 0.0993) (Figure 2E). These results suggest that the density of active genes in a chromosome does not account for the chromosomal activity during ZGA, nor does it account for the utilization of the L and S subgenomes.

### 3.3 Landscape of regional chromosomal activity during ZGA

**FIGURE 3:**
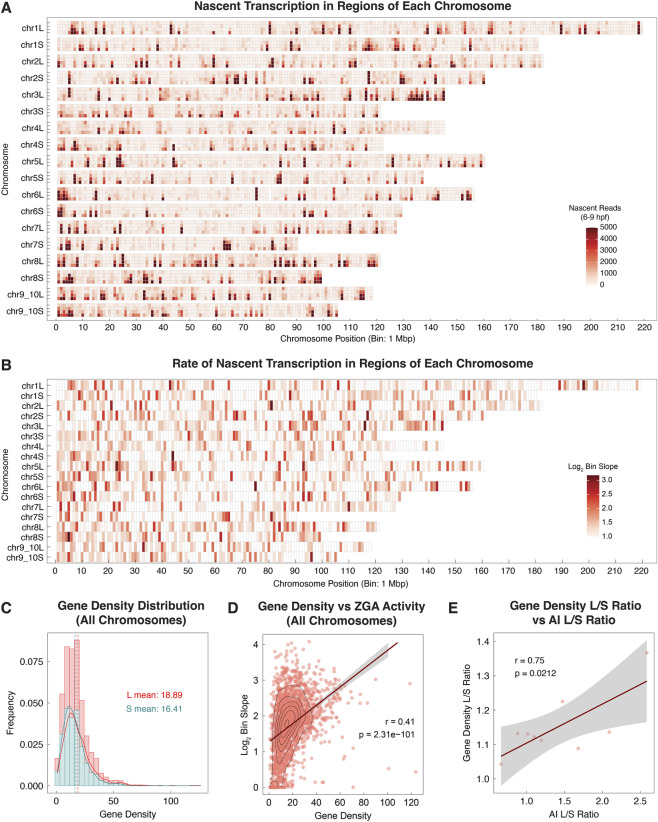
Landscape of regional chromosomal activity during ZGA. **(A)** Heatmap for the net increase in nascent transcript reads from 6-9 hpf (normalized to 5 hpf) for chromosomal regions binned at 1 Mbp. **(B)** Rate of nascent transcription in regions shown in **(A)**. The data were fitted with a linear model and the log_2_ bin slope is shown for each binned region. **(C)** Histogram showing the distribution of gene densities for all chromosomes within bins of 1 million base pair (Mbp). Red and blue indicate L and S chromosomes, respectively. The smooth distribution represents the kernel density estimate for the histogram. The dashed vertical lines indicate the mean density of the distribution, which are annotated in the plots. Chr, chromosome. **(D)** Scatter plot showing correlation between gene density and log_2_ bin slope for all chromosomes. Each dot represents each bin. The data were fitted with a linear regression and shown in dark red, with confidence intervals shown in grey ribbons. The Pearson’s correlation coefficients (r) and p values (p) are labeled. The black contour lines represent the kernel density of the distribution. Chr, chromosome. **(E)** Scatter plot showing correlation between gene density L/S ratio and AI L/S ratio. Each dot represents each chromosome. The data were fitted with a linear regression and shown in dark red, with confidence intervals shown in grey ribbons. The Pearson’s correlation coefficients (r) and p values (p) are labeled.

Our data above suggest that differential activities of chromosomal regions may be important for ZGA. We wondered whether patterns in regional activation of chromosomes exist, and whether they are related to differential and asymmetrical subgenome activation. To investigate this, we calculated the total level of nascent transcripts within every 1 Mbp binned region, at each ZGA stage, and compared the dynamics and rate of ZGA in various regions of individual chromosomes. Remarkably, we found distinct regional activities within all chromosomes, although the degree of difference varies between chromosomes (Figures 3A,B). We also noticed that the gene density in each bin varies dramatically between L and S chromosomes, with L chromosomes having higher gene density than S chromosomes (Figure 3C; Supplementary Figure S3). To identify whether the gene density is related to the regional ZGA activity, we performed the correlation analyses on the number of genes in each 1 Mbp bins and the Log_2_ Bin Slope, a proxy for the regional transcriptional rate. Overall, we found a moderate positive correlation between the gene density and Log_2_ Bin Slope for all chromosomal bins (r = 0.41, p = 2.31e-101) (Figure 3D), although individual chromosomal analysis showed a varying range of correlation between chromosomes (Supplementary Figure S4). Further, to characterize whether the gene density is related to the biased subgenome utilization, we examined the correlation between the gene density L/S ratio and the AI L/S ratio. Strikingly, we found that the gene density L/S ratio and the AI L/S ratio are highly correlated (r = 0.75, p = 0.0212) (Figure 3E). Together, these results suggest that the gene density alone may be an important factor for differential genome activation and biased subgenome utilization during ZGA.

### 3.4 Protein translation regulates asymmetrical subgenome utilization

**FIGURE 4:**
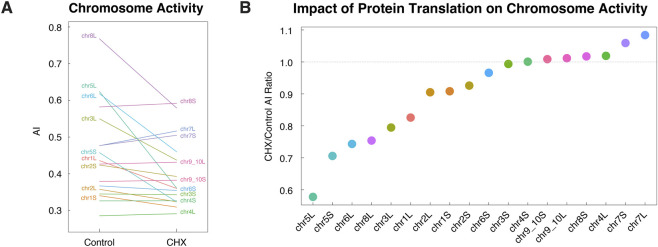
Protein translation regulates asymmetrical subgenome utilization. **(A)** Paired line plot comparing the activation index for each chromosome in control embryos and embryos treated with cycloheximide (CHX), from 5 to 7.5 hpf. **(B)** Dot plot showing the CHX/Control AI ratio for each chromosome. Data ordered by the increasing CHX/Control fold change.

Next, we wondered how the L chromosome-biased utilization is regulated during ZGA. The currently accepted paradigm suggests that ZGA requires translational accumulation of transcriptional activators and lengthening of the cell cycle to initiate transcription (Lee et al., 2014; Schulz and Harrison, 2019; Kojima et al., 2025). We previously found that short treatment of *Xenopus* embryos with cycloheximide (CHX), a protein translation inhibitor, from 5 hpf (pre-ZGA) to 7.5 hpf (ZGA) triggered precocious ZGA (Chen and Good, 2022); however, CHX-induced ZGA may be due to its arresting the embryo and lengthening the cell cycle (Miake-Lye et al., 1983; Gerhart et al., 1984; Newport and Kirschner, 1984; Kimelman et al., 1987), while the impact on chromosome-specific activities remains unknown. To understand whether protein translation regulates L chromosome-biased utilization in ZGA, we analyzed the AI for each chromosome using nascent transcriptome data from control embryos and embryos treated with CHX (Chen and Good, 2022) (Supplementary Figure S5). Intriguingly, CHX specifically reduced the activities of several L-chromosomes, including chr5L, chr6L, chr8L, chr3L, and chr1L, without significantly affecting other chromosomes except for chr5S (Figures 4A,B). These specific CHX-inhibited L chromosomes are among the most active chromosomes with the most biased utilization (Figure 1; Supplementary Figure S1), suggesting that the biased subgenomic utilization during ZGA is primarily regulated by protein translation. Together, these results suggest that maternal translation controls differential chromosome utilization in early embryogenesis, while cell cycle lengthening regulates total transcriptional output during ZGA (Chen and Good, 2022).

## 4 Discussion

In the present study, our activation index (AI) analysis using the temporal nascent transcriptome in *Xenopus* discovers a differential activation and asymmetrical utilization of chromosomes which shapes a landscape of heterogenous ZGA in early development. Our data revealed that ZGA activity is spatially heterogeneous within chromosomes and organized into contiguous high-activity domains, with L chromosomes exhibiting territories with higher gene density than S chromosomes. Such spatial territorial organization cannot be resolved by conventional gene-level expression analyses. Furthermore, our data revealed that maternal translation inhibition selectively suppresses the AI of specific L chromosomes without equivalently affecting their S counterparts or other chromosomes, demonstrating that the maternal translational products amplify the ZGA output of specific chromosomal territories as units, not merely the expression of individual ZGA genes on those chromosomes. Previous studies have shown heterogenous zygotic gene activation at the level of single genes (Stapel et al., 2017), single cells (Chen et al., 2019), and single alleles (Wei et al., 2024); however, the single chromosome level has previously been unexplored. Recent studies suggest a divergent activation of the homoeologous genes from the L and S subgenomes of the allotetroploid *Xenopus laevis* early embryos (Session et al., 2016; Phelps et al., 2023). Our finding that the differential chromosomal activity biased towards L chromosomes is consistent with this. Notably, pluripotency factors Pou5f3 and Sox3 activate the L and S subgenomes differently during ZGA, resulting in asymmetric activation of gene pairs (Phelps et al., 2023). Our study revealed that protein translation is required for regulating the asymmetrical chromosome utilization during ZGA, and mediation by the potential translational accumulation of Pou5f3 and Sox3 in early embryos merits further investigation. Further, due to the important role of chromatin accessibility in regulating early embryonic gene expression (Gentsch et al., 2019), it requires further study whether these pluripotency factors mediate the intra-chromosomal heterogeneity in zygotic transcription, which also varies significantly between chromosomes. Moreover, whether the highly active regions on different chromosomes are co-regulated is unknown, and it would be intriguing to characterize the higher-order three-dimensional regional interactions among the subgenomes (Hug and Vaquerizas, 2018; Vallot and Tachibana, 2020; Sivkina et al., 2025).

In summary, our work provides a new framework of biased genome activation during early embryogenesis at the chromosomal level. The asymmetrical utilization of chromosomes for ZGA may be conserved in other species.

## Data Availability

Publicly available datasets analyzed in this work are available in Gene Expression Omnibus (GEO) https://www.ncbi.nlm.nih.gov/geo/. The nascent transcriptome data from *Xenopus laevis* embryos at 5-9 hours post-fertilization (hpf) are part of the subseries of RNA-seq datasets GSE201835 (GSM6076974, GSM6076975, GSM6076976, GSM6076977, GSM6076978, GSM6076979, GSM6076980, GSM6076981, GSM6076982, and GSM6076983). The nascent transcriptome data from control- and cycloheximide (CHX)-treated *Xenopus laevis* embryos at 5-7.5 hpf are part of the subseries of RNA-seq datasets GSE201834 (GSM6076934, GSM6076935, GSM6076936, GSM6076937, GSM6076938, GSM6076939).

## References

[B1] Bhat P. Cabrera-Quio L. E. Herzog V. A. Fasching N. Pauli A. Ameres S. L. (2023). SLAMseq resolves the kinetics of maternal and zygotic gene expression during early zebrafish embryogenesis. Cell Rep. 42 (2), 112070. 10.1016/j.celrep.2023.112070 36757845

[B2] Chan S. H. Tang Y. Miao L. Darwich-Codore H. Vejnar C. E. Beaudoin J. D. (2019). Brd4 and P300 confer transcriptional competency during zygotic genome activation. Dev. Cell 49 (6), 867–881 e868. 10.1016/j.devcel.2019.05.037 31211993 PMC7201981

[B3] Chen H. (2025). Quantifying nascent transcription in early embryogenesis. Methods Mol. Biol. 2923, 143–162. 10.1007/978-1-0716-4522-2_9 40418448 PMC12181038

[B4] Chen H. Good M. C. (2022). Nascent transcriptome reveals orchestration of zygotic genome activation in early embryogenesis. Curr. Biol. 32 (19), 4314–4324 e4317. 10.1016/j.cub.2022.07.078 36007528 PMC9560990

[B5] Chen H. Einstein L. C. Little S. C. Good M. C. (2019). Spatiotemporal patterning of zygotic genome activation in a model vertebrate embryo. Dev. Cell 49 (6), 852–866 e857. 10.1016/j.devcel.2019.05.036 31211992 PMC6655562

[B6] Gentsch G. E. Spruce T. Owens N. D. L. Smith J. C. (2019). Maternal pluripotency factors initiate extensive chromatin remodelling to predefine first response to inductive signals. Nat. Commun. 10 (1), 4269. 10.1038/s41467-019-12263-w 31537794 PMC6753111

[B7] Gerhart J. Wu M. Kirschner M. (1984). Cell cycle dynamics of an M-phase-specific cytoplasmic factor in *Xenopus laevis* oocytes and eggs. J. Cell Biol. 98 (4), 1247–1255. 10.1083/jcb.98.4.1247 6425302 PMC2113233

[B8] Heyn P. Kircher M. Dahl A. Kelso J. Tomancak P. Kalinka A. T. (2014). The earliest transcribed zygotic genes are short, newly evolved, and different across species. Cell Rep. 6 (2), 285–292. 10.1016/j.celrep.2013.12.030 24440719

[B9] Hug C. B. Vaquerizas J. M. (2018). The birth of the 3D genome during early embryonic development. Trends Genet. 34 (12), 903–914. 10.1016/j.tig.2018.09.002 30292539

[B10] Jukam D. Shariati S. A. M. Skotheim J. M. (2017). Zygotic genome activation in vertebrates. Dev. Cell 42 (4), 316–332. 10.1016/j.devcel.2017.07.026 28829942 PMC5714289

[B11] Kimelman D. Kirschner M. Scherson T. (1987). The events of the midblastula transition in xenopus are regulated by changes in the cell cycle. Cell 48 (3), 399–407. 10.1016/0092-8674(87)90191-7 3802197

[B12] Kojima M. L. Hoppe C. Giraldez A. J. (2025). The maternal-to-zygotic transition: reprogramming of the cytoplasm and nucleus. Nat. Rev. Genet. 26 (4), 245–267. 10.1038/s41576-024-00792-0 39587307 PMC11928286

[B13] Kwasnieski J. C. Orr-Weaver T. L. Bartel D. P. (2019). Early genome activation in drosophila is extensive with an initial tendency for aborted transcripts and retained introns. Genome Res. 29 (7), 1188–1197. 10.1101/gr.242164.118 31235656 PMC6633261

[B14] Lee M. T. Bonneau A. R. Giraldez A. J. (2014). Zygotic genome activation during the maternal-to-zygotic transition. Annu. Rev. Cell Dev. Biol. 30, 581–613. 10.1146/annurev-cellbio-100913-013027 25150012 PMC4303375

[B15] Li L. Lu X. Dean J. (2013). The maternal to zygotic transition in mammals. Mol. Asp. Med. 34 (5), 919–938. 10.1016/j.mam.2013.01.003 23352575 PMC3669654

[B16] Love M. I. Huber W. Anders S. (2014). Moderated estimation of fold change and dispersion for RNA-Seq data with DESeq2. Genome Biol. 15 (12), 550. 10.1186/s13059-014-0550-8 25516281 PMC4302049

[B17] Miake-Lye R. Newport J. Kirschner M. (1983). Maturation-promoting factor induces nuclear envelope breakdown in cycloheximide-arrested embryos of *Xenopus laevis* . J. Cell Biol. 97 (1), 81–91. 10.1083/jcb.97.1.81 6345556 PMC2112507

[B18] Newport J. W. Kirschner M. W. (1984). Regulation of the cell cycle during early xenopus development. Cell 37 (3), 731–742. 10.1016/0092-8674(84)90409-4 6378387

[B19] Patro R. Duggal G. Love M. I. Irizarry R. A. Kingsford C. (2017). Salmon provides fast and bias-aware quantification of transcript expression. Nat. Methods 14 (4), 417–419. 10.1038/nmeth.4197 28263959 PMC5600148

[B20] Phelps W. A. Hurton M. D. Ayers T. N. Carlson A. E. Rosenbaum J. C. Lee M. T. (2023). Hybridization led to a rewired pluripotency network in the allotetraploid *Xenopus laevis* . Elife 12, e83952. 10.7554/eLife.83952 37787392 PMC10569791

[B21] Schier A. F. (2007). The maternal-zygotic transition: death and birth of RNAs. Science 316 (5823), 406–407. 10.1126/science.1140693 17446392

[B22] Schulz K. N. Harrison M. M. (2019). Mechanisms regulating zygotic genome activation. Nat. Rev. Genet. 20 (4), 221–234. 10.1038/s41576-018-0087-x 30573849 PMC6558659

[B23] Session A. M. Uno Y. Kwon T. Chapman J. A. Toyoda A. Takahashi S. (2016). Genome evolution in the allotetraploid frog *Xenopus laevis* . Nature 538 (7625), 336–343. 10.1038/nature19840 27762356 PMC5313049

[B24] Sivkina A. L. Iarovaia O. V. Razin S. V. Ulianov S. V. (2025). The establishment of the 3D genome structure during zygotic genome activation. Ann. N. Y. Acad. Sci. 1545 (1), 38–51. 10.1111/nyas.15304 40029160

[B25] Skirkanich J. Luxardi G. Yang J. Kodjabachian L. Klein P. S. (2011). An essential role for transcription before the MBT in *Xenopus laevis* . Dev. Biol. 357 (2), 478–491. 10.1016/j.ydbio.2011.06.010 21741375 PMC3164747

[B26] Stapel L. C. Zechner C. Vastenhouw N. L. (2017). Uniform gene expression in embryos is achieved by temporal averaging of transcription noise. Genes Dev. 31 (16), 1635–1640. 10.1101/gad.302935.117 28903980 PMC5647934

[B27] Tadros W. Lipshitz H. D. (2009). The maternal-to-zygotic transition: a play in two acts. Development 136 (18), 3033–3042. 10.1242/dev.033183 19700615

[B28] Vallot A. Tachibana K. (2020). The emergence of genome architecture and zygotic genome activation. Curr. Opin. Cell Biol. 64, 50–57. 10.1016/j.ceb.2020.02.002 32220807 PMC7374442

[B29] Vastenhouw N. L. Cao W. X. Lipshitz H. D. (2019). The maternal-to-zygotic transition revisited. Development 146 (11). 10.1242/dev.161471 31189646

[B30] Wei J. Zhang W. Jiang A. Peng H. Zhang Q. Li Y. (2024). Temporospatial hierarchy and allele-specific expression of zygotic genome activation revealed by distant interspecific urochordate hybrids. Nat. Commun. 15 (1), 2395. 10.1038/s41467-024-46780-0 38493164 PMC10944513

[B31] Wu E. Vastenhouw N. L. (2020). From mother to embryo: a molecular perspective on zygotic genome activation. Curr. Top. Dev. Biol. 140, 209–254. 10.1016/bs.ctdb.2020.02.002 32591075

[B32] Zhou C. Y. Heald R. (2023). Principles of genome activation in the early embryo. Curr. Opin. Genet. Dev. 81, 102062. 10.1016/j.gde.2023.102062 37339553 PMC11419330

