# Supplementary material for: Asymmetrical chromosomal utilization during zygotic genome activation
Source: Front Bioinform. 2026 Jul 1;6:1851810. doi: 10.3389/fbinf.2026.1851810 (PMC13368737; doi:10.3389/fbinf.2026.1851810)
Supplement: Supplementary file 1 [file DataSheet1.PDF]

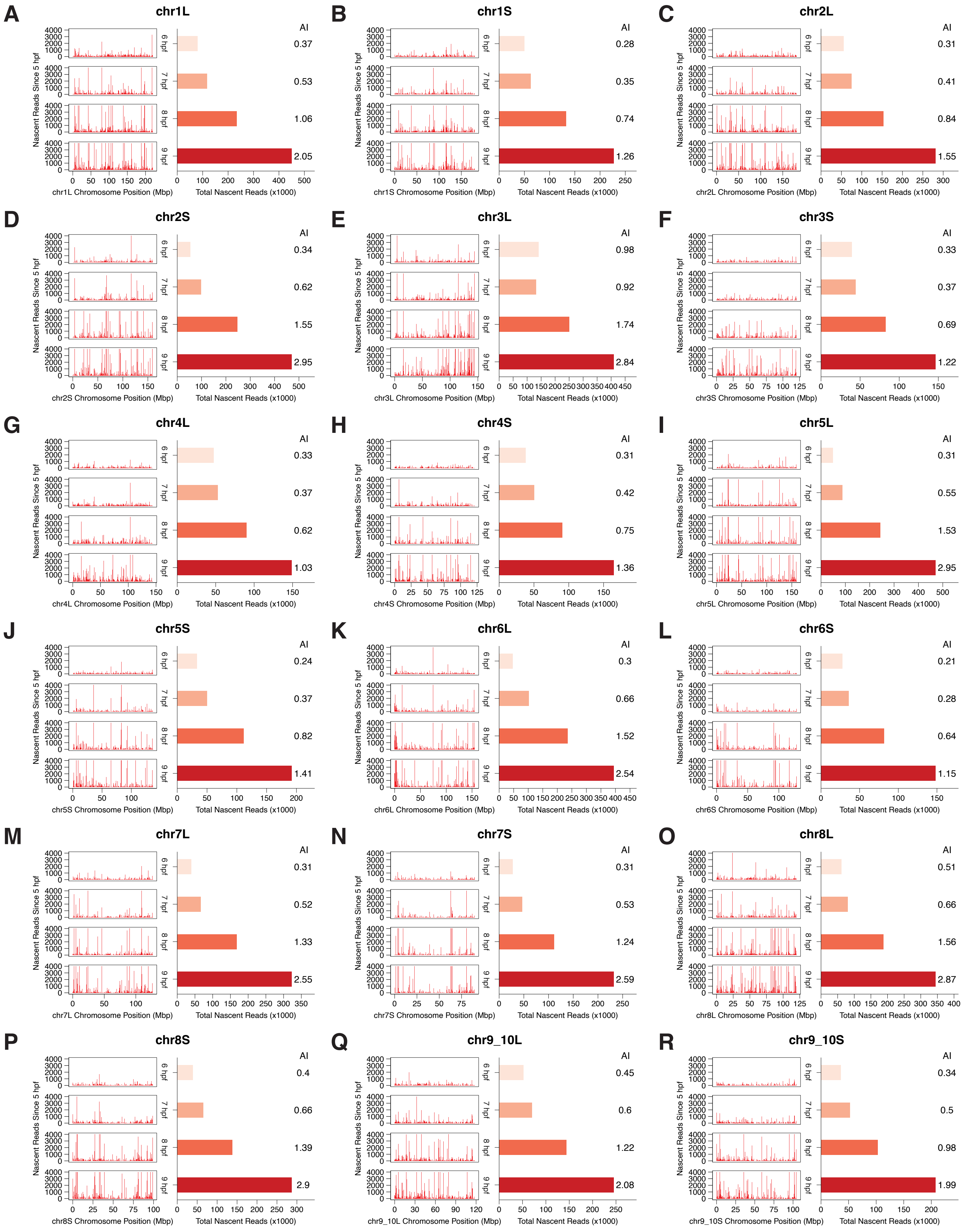

Figure. S1 Chromosomal Activities during Zygotic Genome Activation. (A-R) Spike plots for the net increase in nascent transcript reads for individual genes (Left) and bar plots showing the total net nascent transcript reads (Right) from 6-9 hpf normalized to 5 hpf on each chromosome. The activation index (AI) shown is represented as the net increase in nascent transcript reads per kilo base for each chromosome. chr, chromosome; Mbp, million base pair; hpf, hours post-fertilization.

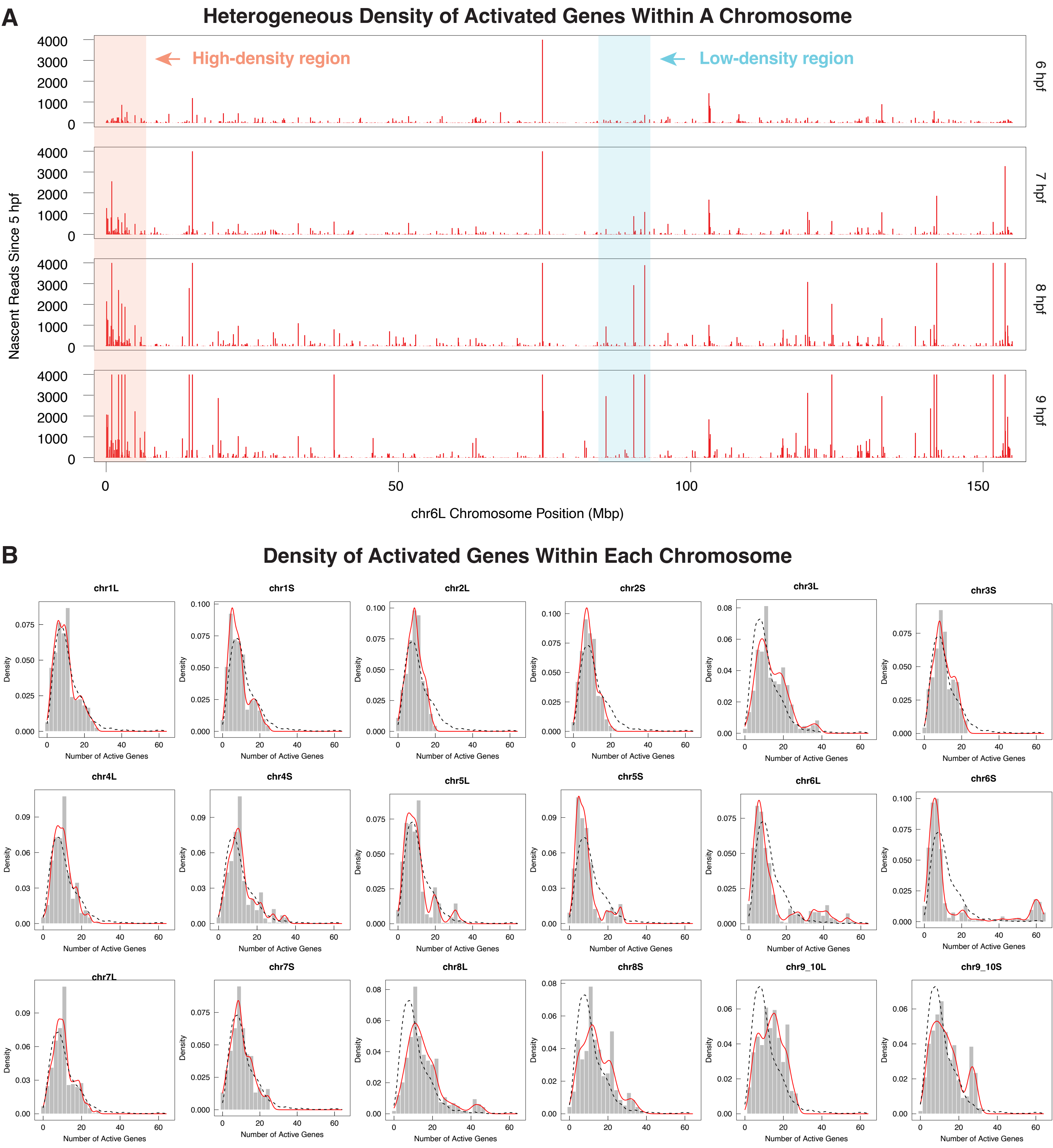

Figure. S2 Heterogeneous Transcription in Chromosome. (A) Example of high-density region and low-density region for transcription on chromosome 6L (chr6L). (B) Density of activated genes within a range of 1 million base pair (Mbp) from a gene on each chromosome during ZGA from 5 to 9 hours post-fertilization (hpf). The gene activity was determined by fitting a linear model and the genes with a slope > 1 are included. The red lines indicate the density of activated genes in respective chromosomes, while the dashed black lines indicate the density of activated genes across all chromosomes. chr, chromosome; Mbp, million base pair; hpf, hours post-fertilization.

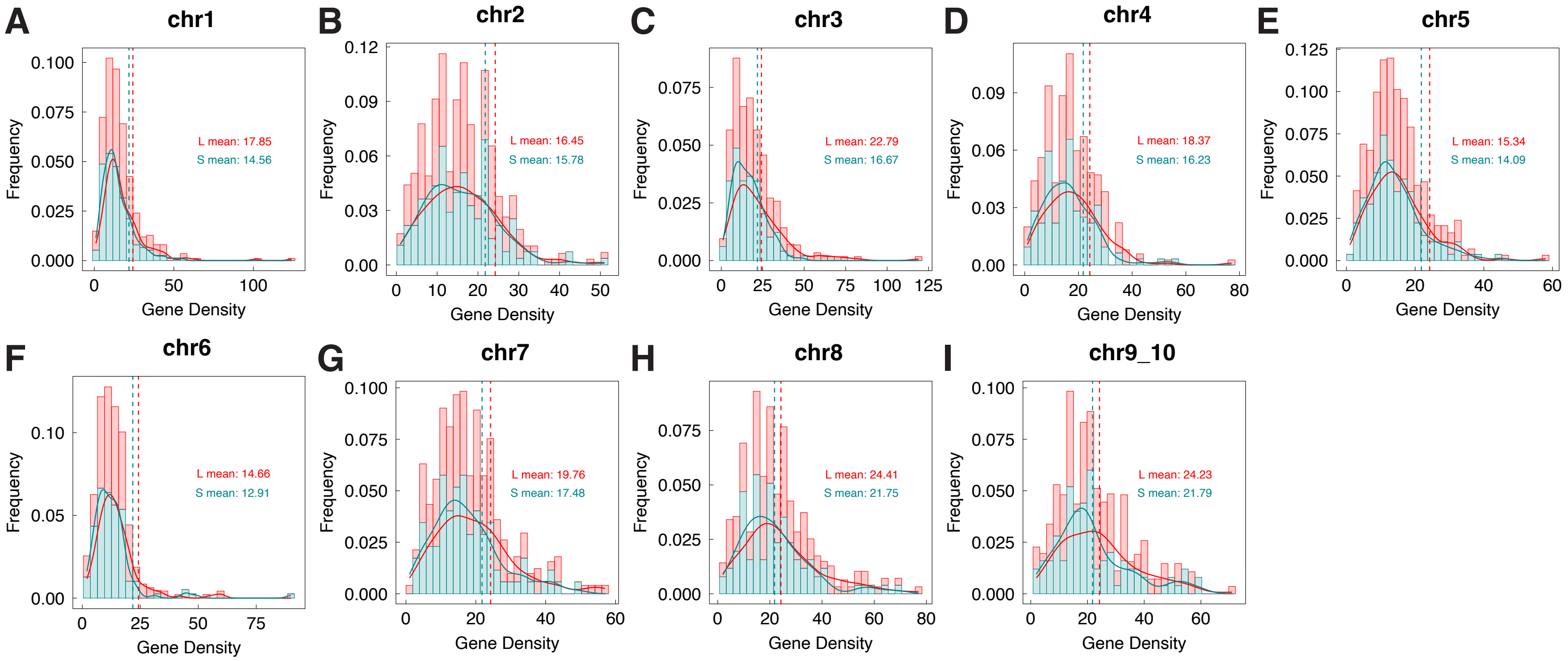

Figure. S3 Distribution of Gene Density in Individual Chromosomes. (A-I) Histograms showing the distribution of gene densities for each of chromosomes within bins of 1 million base pair (Mbp). The gene density is represented by the numbers of genes in each bin of individual chromosomes. Red and blue indicate L and S chromosomes, respectively. The smooth distribution represents the kernel density estimate for the histogram. The dashed vertical lines indicate the mean density of the distribution, which are annotated in the plots. chr, chromosome.

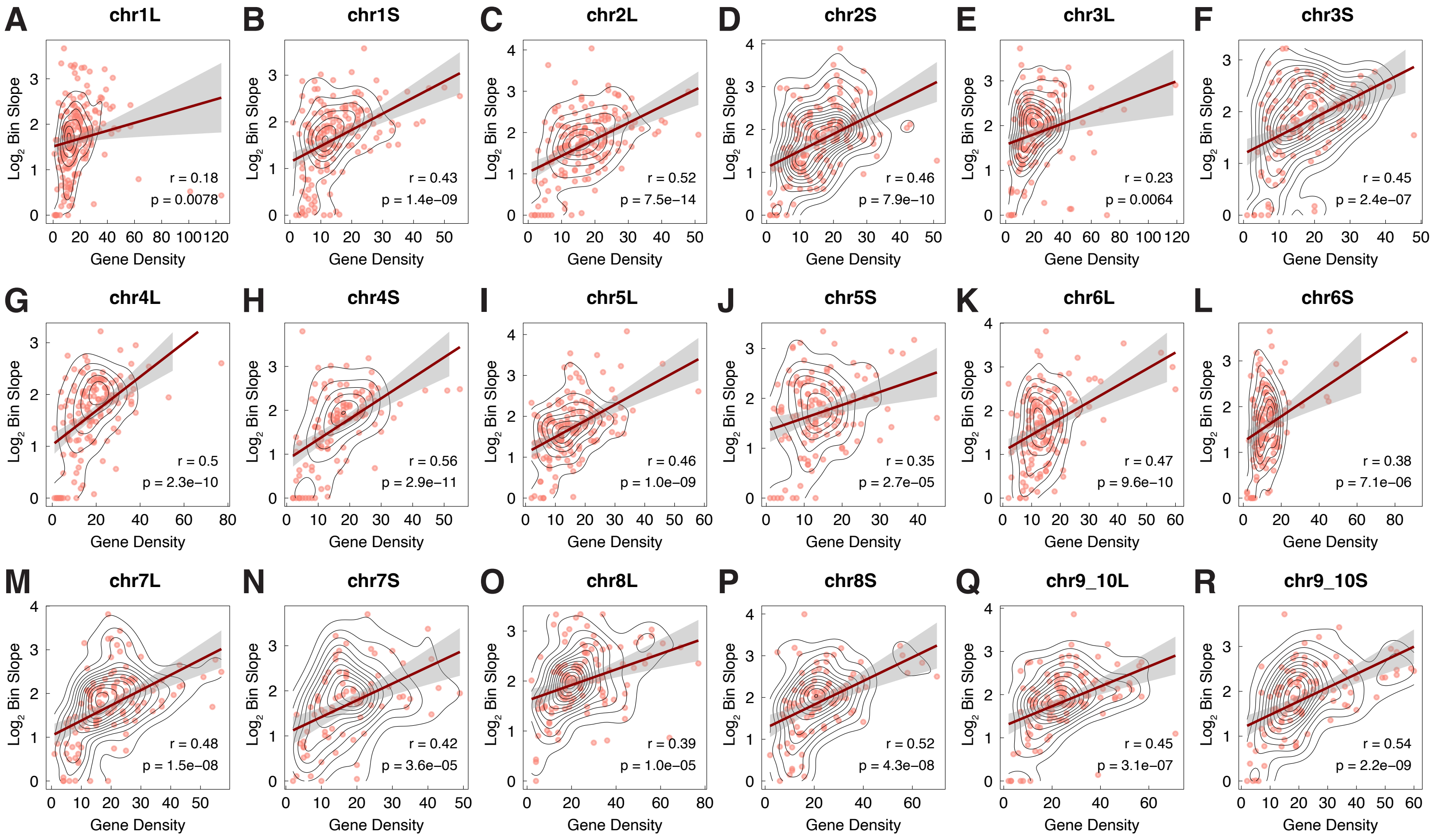

Figure. S4 Correlation Between Gene Density and Regional ZGA Activity in Individual Chromosomes. (A-R) Scatter plots showing correlation between gene density and $\log_2$ bin slope. Each chromosome was binned at an interval of 1 million base pair (Mbp). The gene density is represented by the numbers of genes in each bin of individual chromosomes, and the $\log_2$ bin slope was determined after fitting a linear model for the net increased nascent transcripts during ZGA from 5 to 9 hours post-fertilization (hpf) within each bin. Each dot represents each bin. The data were fitted with a linear regression and shown in dark red, with confidence intervals shown in grey ribbons. The Pearson's correlation coefficients (r) and p values (p) are labeled. The black contour lines represent the kernel density of the distribution. chr, chromosome.

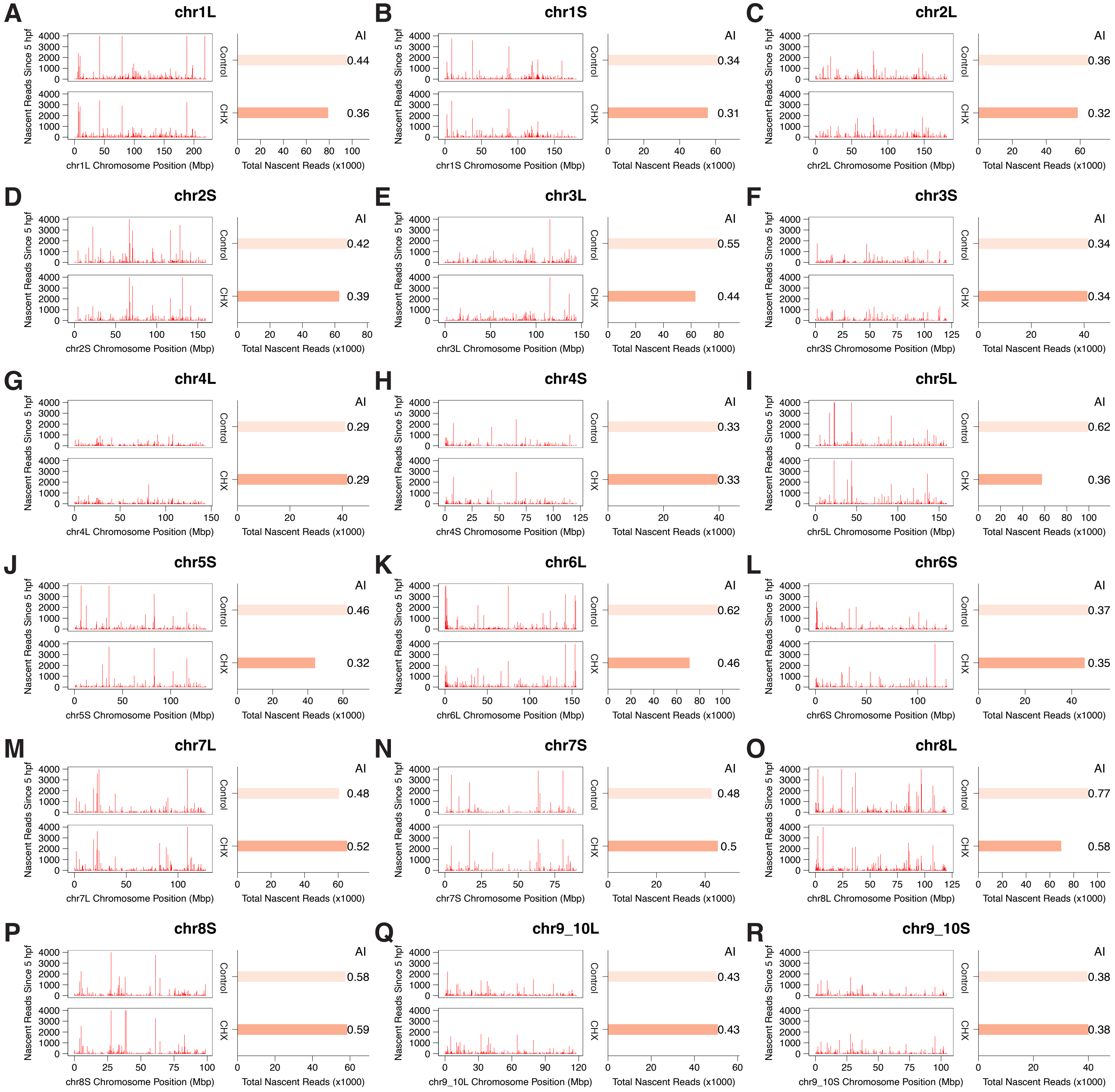

Figure. S5 Effect of Cycloheximide on Chromosome Activity. (A-R) Spike plots for the net increase in nascent transcript reads for individual genes (Left) and bar plots showing the total net nascent transcript reads (Right) in control embryos and embryos treated with cycloheximide (CHX) from 5-7.5 hpf. The activation index (AI) shown is represented as the net increase in nascent transcript reads per kilo base for each chromosome. chr, chromosome; Mbp, million base pair; hpf, hours post-fertilization.
